# The N‐terminal domains of the paralogous HycE and NuoCD govern assembly of the respective formate hydrogenlyase and NADH dehydrogenase complexes

**DOI:** 10.1002/2211-5463.12787

**Published:** 2020-02-04

**Authors:** Philipp Skorupa, Ute Lindenstrauß, Sabrina Burschel, Christian Blumenscheit, Thorsten Friedrich, Constanze Pinske

**Affiliations:** ^1^ Institute of Biology/Microbiology Martin‐Luther University Halle‐Wittenberg Germany; ^2^ Institute of Biochemistry/Molecular Bioenergetics Albert‐Ludwigs‐University Freiburg Germany

**Keywords:** complex I, formate hydrogenlyase, fusion proteins, HycE, NADH:ubiquinone oxidoreductase, NuoCD

## Abstract

Formate hydrogenlyase (FHL) is the main hydrogen‐producing enzyme complex in enterobacteria. It converts formate to CO_2_ and H_2_ via a formate dehydrogenase and a [NiFe]‐hydrogenase. FHL and complex I are evolutionarily related and share a common core architecture. However, complex I catalyses the fundamentally different electron transfer from NADH to quinone and pumps protons. The catalytic FHL subunit, HycE, resembles NuoCD of *Escherichia coli* complex I; a fusion of NuoC and NuoD present in other organisms. The C‐terminal domain of HycE harbours the [NiFe]‐active site and is similar to other hydrogenases, while this domain in NuoCD is involved in quinone binding. The N‐terminal domains of these proteins do not bind cofactors and are not involved in electron transfer. As these N‐terminal domains are separate proteins in some organisms, we removed them in *E. coli* and observed that both FHL and complex I activities were essentially absent. This was due to either a disturbed assembly or to complex instability. Replacing the N‐terminal domain of HycE with a 180 amino acid *E. coli* NuoC protein fusion did not restore activity, indicating that the domains have complex‐specific functions. A FHL complex in which the N‐ and C‐terminal domains of HycE were physically separated still retained most of its FHL activity, while the separation of NuoCD abolished complex I activity completely. Only the FHL complex tolerates physical separation of the HycE domains. Together, the findings strongly suggest that the N‐terminal domains of these proteins are key determinants in complex assembly.

## Graphical abstract

Complex I and the formate hydrogenlyase (FHL) complex from *Escherichia coli* contain either the fusion protein NuoCD or HycE, respectively. We deleted the N‐terminal, cofactor‐free domains from both proteins or genetically separated them into two individual proteins and monitored enzyme activity. The deletion caused instability of both complexes, while after separation, FHL retained activity but complex I was inactive.

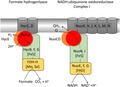

**Abbreviations**
BV: benzyl viologen
FdhH: formate dehydrogenase
FHL: formate hydrogenlyase
Nuo: NADH:ubiquinone oxidoreductase

Protein tertiary structure describes the autonomously folding regions of a protein as a domain. Domains can serve different functions, for example binding of cofactors, recognition of a motif or catalytic activity. Evolution has shuffled domains to create the huge diversity of proteins that occur today 1. Taken on their own, a domain sometimes behaves completely different compared to the holo‐protein, as observed for fibril formation by the isolated N‐terminal acylphosphatase domain of the HypF hydrogenase maturation factor 2, 3. Artificial fusion proteins have been used for many years as biochemical tools, and in many cases, these fusions have no or little impact on the function of the original protein.

It has been observed that proteins possessing coordinate functions sometimes become fused during evolution, allowing greater efficiency, based on colocation. One prominent example is the alcohol dehydrogenase fusion with acetaldehyde dehydrogenase, which couples two sequential reactions without releasing the toxic intermediate acetaldehyde 4, 5. The most straightforward way for these fusion events to occur is when neighbouring genes of a polycistronic operon are joined through frameshift mutations. Clearly, evolutionary selective pressure is required for the proteins to remain fused, for example when the activity is enhanced or assembly is augmented. Protein complexes with several subunits are likely to assemble via ordered pathways, and natural selection tends to favour gene fusions to optimize assembly 6. Gene fusion seems also to have happened to the *hycE* gene of the *Escherichia coli* hydrogenase three large subunit, HycE. Together with a formate dehydrogenase (FdhH), the HycE hydrogenase forms the catalytic components of the formate hydrogenlyase (FHL) complex in *E. coli* and other Enterobacteriacea. FHL catalyses the oxidation of formate with the concomitant reduction of protons to produce hydrogen to detoxify formate during mixed‐acid fermentation 7. Along with the FdhH and HycE, three iron‐sulfur proteins, including the small subunit HycG, HycF and HycB, form the soluble part of the FHL complex. This large soluble domain is anchored to the membrane by two membrane subunits 8. The complex is phylogenetically and structurally related to the respiratory complex I, with the NuoCD protein being the homologue of HycE 9, 10, 11, 12. Although the relationship is less obvious on the primary structural level, where HycE and NuoCD share only 26% (145/563) identical and 43% (244/563) similar residues, it is clearly apparent when the secondary structural elements are compared between the two proteins (Fig. 1). Complex I, or NADH:ubiquinone oxidoreductase (Nuo), is the primary energy‐conserving complex of many respiratory chains and couples NADH oxidation to the translocation of four protons across the membrane 13. NuoC varies significantly in length in different organisms 14. Unlike most organisms, which have separate NuoC and NuoD proteins, both are fused in complex I of *E. coli* and *Bacteroides fragilis*. Moreover, the NuoC subunit is homologous to the N‐terminal domain of HycE and its paralogue in *E. coli*, HyfG 14. The crystal structures of both the soluble portion and the entire complex from *Thermus thermophilus* have been determined 15, 16. NuoC is an orthologue of Nqo5 in *T. thermophilus* and of the 30‐kDa protein in *Bos taurus* 13. Unlike its counterpart in HycE, which contains the [NiFe]‐active site, NuoD is not known to harbour any cofactors, but it does have a quinone‐binding site 17.

**Figure 1:**
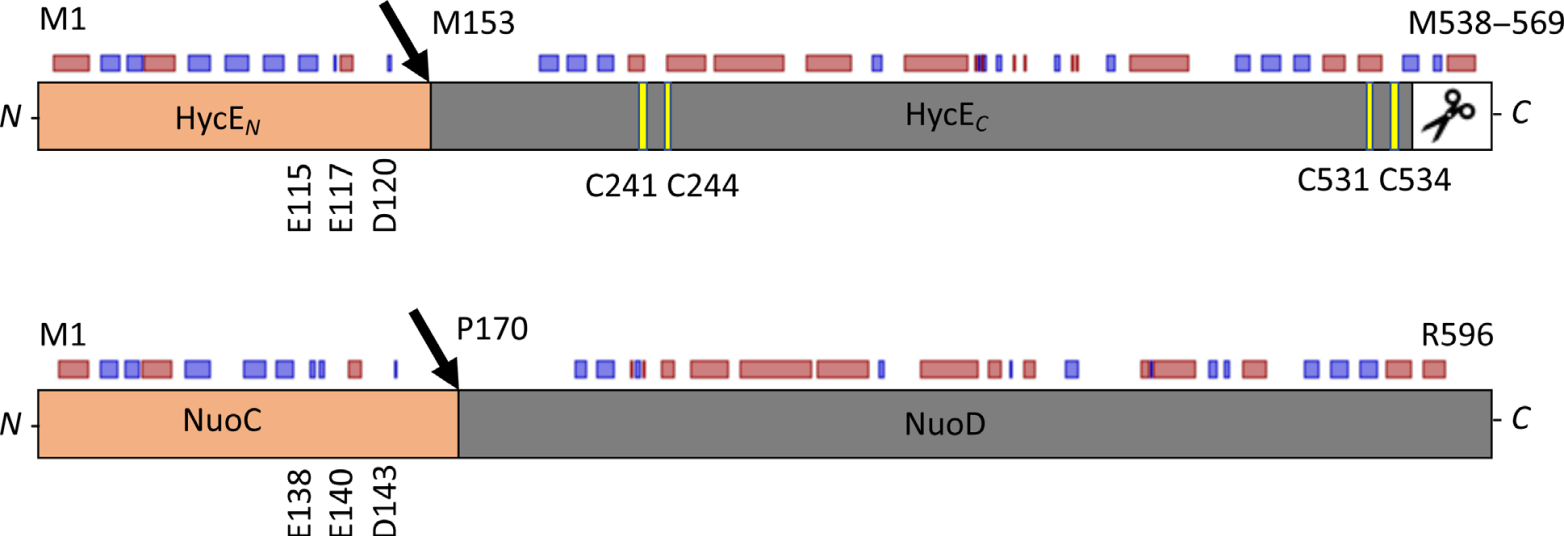
Schematic representation of the HycE (top) and NuoCD (bottom) fusion proteins. The N‐terminal domain of each protein is coloured in orange and the C‐terminal domain in grey, and the black arrows indicate the sites where the proteins were genetically separated. The cysteinyl residues that coordinate the [NiFe]‐cofactor in HycE are coloured in yellow, and the C‐terminal extension that is proteolytically processed is shown in white with the scissors symbol. The locations of further relevant residues, which are mentioned in the text, are shown. The small rectangles above the respective proteins represent the secondary structure prediction (red – helix; blue – sheet) based on the Open PredictProtein server 51.

The C‐terminal domain of HycE extends from amino acids 175–537 and harbours the four conserved cysteinyl residues that coordinate the bimetallic [NiFe]‐cofactor required for catalysis (scheme in Fig. 1 and alignment in Fig. 2). These cysteinyl residues are not present in NuoD (Figs 1 and 2). Six pleiotropic Hyp proteins are required for synthesis of the [NiFe]‐cofactor including its diatomic ligands attached to the Fe atom 18, 19. Of these proteins, the HypC and HypA proteins interact directly with HycE for Fe(CN)_2_CO and Ni^2+^ insertion, respectively 20, 21. In addition, HycE has a 32‐amino acid C‐terminal extension from amino acids 538–569 that is endoproteolytically processed after cofactor insertion and is assumed to facilitate this step by keeping the empty, cofactor‐free apo‐protein in an open conformation 22. In this context, the N terminus of the unprocessed hydrogenase large subunit from *Thermococcus kodakarensis* was found to extend into the nickel‐delivery protein HypA, while the C‐terminal extension replaces the position of the N terminus prior to proteolytic processing 23. A recent DFT calculation on HycE identified conserved glutamate residues in both the N‐ and C‐terminal domains, which are proposed to govern the insertion of the Ni^2+^ ion of the cofactor, but the function of these residues has yet to be validated experimentally for HycE 24. This strongly suggests that both domains act in concert for cofactor insertion.

**Figure 2:**
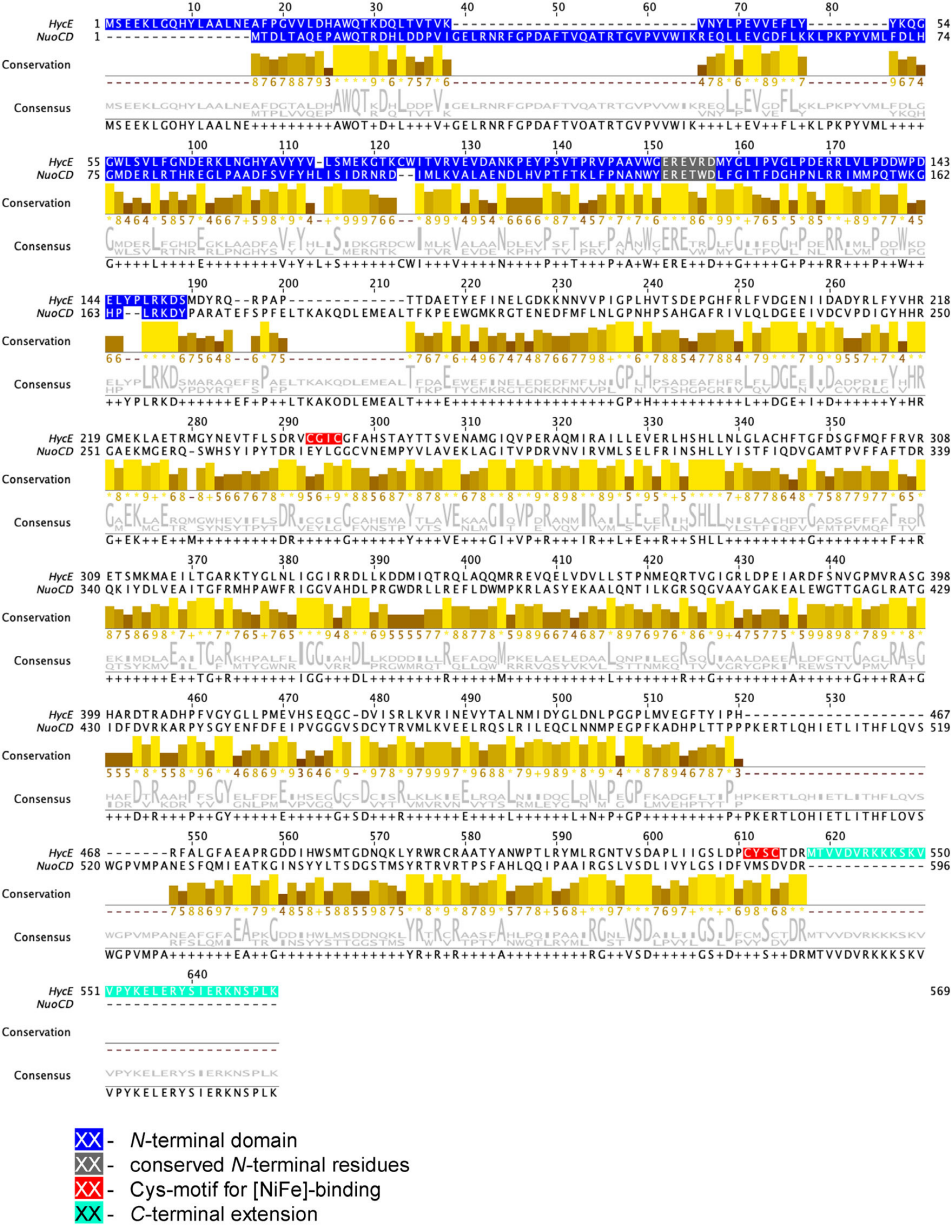
Alignment of HycE and NuoCD protein sequences. The alignment was performed with clustal o (1.2.4) 55 within the UniProt database and the visualization done by jalview 52; in the conservation panel, the height of the bar represents the conservation as well as the consensus logo provided according to the size of the letter. Highlighted within the sequences are the N‐terminal domains (blue highlight), conserved residues within the N‐terminal domain (grey highlight), hydrogenase specific C‐terminal extension (cyan highlight) and [NiFe]‐cofactor binding motif (red highlight).

The N‐terminal domain of HycE, corresponding to NuoC, extends from amino acids 9–194 and thus is predicted to have a short overlap with the C‐terminal domain 25. This domain/protein bears a cofactor neither in the FHL complex nor in complex I, and its role remains enigmatic.

The N‐ and C‐terminal domains of HycE and NuoCD are predicted to be fused via a long unstructured loop region that contacts the formate dehydrogenase or diaphorase subunits in FHL and complex I, respectively (Fig. 1). Previous work has shown that conserved residues (Glu‐138, Glu‐140, Asp‐143) in the N‐terminal domain NuoC are required for the stability of not only the NuoCD protein, but also all other subunits 26. These residues are also conserved in the HycE protein (Figs 1 and 2). Amino acid exchanges within the N‐terminal domain of HycE were better tolerated than exchanges in these conserved residues; for example, the addition of a 10‐His residue stretch after Gly‐83 was shown to have no effect on the stability and activity of the FHL complex because they are exposed to the surface 27. Moreover, engineering of the HycE protein revealed that truncation of the C terminus increased H_2_ production from the FHL complex, despite the fact that these deletions remove the [NiFe]‐binding site 28.

Therefore, in this study, we investigated the possibility of splitting the HycE and NuoCD domains into separate proteins in order to address the role of the N‐terminal domain and the potential evolutionary significance of the fusion.

## Methods

### Growth conditions

Bacterial growth was generally carried out either aerobically on LB agar plates and in shaking liquid LB cultures or anaerobically as standing liquid cultures in TGYEP medium, pH 6.5, with 0.8% (w/v) glucose included 29 at 37 °C. Alternatively, for complex I activity, aerobic cultures were grown in glycerol medium [1% (w/v) peptone, 0.5% (w/v) yeast extract, 0.8% (w/v) NaCl, 0.4% (w/v) glycerol and separately prepared 15 mm Na_3_PO_4_, 10 mm Na_2_HPO_4_ and 10 mm KH_2_PO_4_, pH 8.5]. Cultures were vigorously shaken at 180 r.p.m. and 37 °C. Growth experiments were performed as biological triplicates in a 96‐well microtiter plate, filled with 150 μL M9 minimal medium with 25 mm acetate as sole carbon source (47.6 mm Na_2_HPO_4_, 22 mm KH_2_PO_4_, 8.4 mm NaCl, 19 mm NH_4_Cl, 2 mm MgSO_4_, 0.1 mm CaCl_2_, 0.3 μm thiamine dichloride, 0.1% w/v trace element solution) and agitation of 150 r.p.m. in a Tecan Infinite 200 plate reader (TECAN, Männedorf, Switzerland). Strains for maintaining the pMAK705 plasmids were grown aerobically at 30 °C. When appropriate, the antibiotics ampicillin and chloramphenicol were included at 100 and 34 μg·mL^−1^, respectively.

### DNA modifications and strain construction

All strains and plasmids are listed in Table 1 and oligonucleotides in Table 2. For constructing the N‐terminal truncation of HycE in strain Δ*hycE*N, the 514‐bp DNA upstream region up to the encoded methionine start codon was amplified using the oligonucleotides HycEup_XbaI and HycEup_BamHI leaving the ribosome binding site intact, while the downstream 1254 bp was amplified from methionine codon 153 to the stop codon using the oligonucleotides HycEdown_BamHI and HycEdown_EcoRI. The two fragments were digested with XbaI/BamHI and BamHI/EcoRI, respectively, and ligated into pBSK(+) before being moved by XbaI/EcoRI restriction digestion to pMAK705 for chromosomal integration. The chromosomal integration into strain MG1655 was performed according to Ref. 30 and yielded the strain Δ*hycE*N. Similarly, the above pMAK705 construct was modified by introducing the *nuoC* DNA sequence into the BamHI site after amplification with the oligonucleotides nuoC_HA_FW_BamHI and nuoC_RW_BamHI. The directionality of the insert was verified by DNA sequencing, and the integration onto the chromosome of MG1655 was done independently and resulted in strain *nuoC‐hycE*.

**Table 1:** Strains and plasmids used in this study.

	Genotype	Reference
Strain		
MG1655	F^−^ λ^−^ *ilvG* ^−^ *rfb‐50 rph‐1*	53
BW25113	*lacI* ^+^ *rrnB*_T14_ Δ*lacZ*_WJ16_ *hsdR*514 Δ*araBAD*_AH33_ Δ*rhaBAD*_LD78_ *rph‐1* Δ*(araB–D)567* Δ*(rhaD–B)568* Δ*lacZ4787*(::*rrnB‐3*) *hsdR514* *rph‐1*	33, 54
JW2691	Like BW25113, but Δ*hycE*	54
*hycE*N+C	Like MG1655, but *hycE* separated as AA1‐152 and AA153‐569	This study
Δ*hycE*N	Like MG1655, but Δ*hycE* N‐terminal domain corresponding to AA1‐152	This study
*nuoC‐hycE*	Like MG1655, but *hycE* N‐terminal domain AA1‐152 replaced with *nuoC* coding for AA1‐169	This study
JW4040	Like BW25113, but Δ*fdhF*	54
Δ*ndh*	JW1095: like BW25113, but Δ*ndh*	31, 54
Δ*nuoC*	BW25113 Δ*ndh*, Δ*nuoC* AA1‐169	This study
*nuoC+D*	BW25113 Δ*ndh*,* nuoC* separated as AA1‐169 and *nuoD* as AA170‐596	This study
Δ*nuoC‐L*	BW25113 Δ*ndh*, Δ*nuoC‐L*	This study
Plasmids		
pMAK705	Temperature‐sensitive replicon, Cm^R^	30
pHycE	pACYCDuet‐1, *hycE* ^+^, internal His‐tag, Cm^R^	34
pHycEN+C	pACYCDuet‐1, *hycE* N‐terminal domain AA1‐152, *hycE* C‐terminal domain AA153‐569 Cm^R^	This study
pHycEN	pJET1.2, *hycE* N‐terminal domain AA1‐168, Amp^R^	This study
pJET‐nuoCD	pJET1.2, *nuoCD* including RBS, Amp^R^	This study
pKD3	Cm^R^	33
pCP20	Contains flippase gene for λ Red mutagenesis; Cm^R^, Amp^R^	32

**Table 2:** Oligonucleotides used in this study.

Oligonucleotide	Sequence 5′ → 3′
HycEup_XbaI	GCGTCTAGATGCTGCTTGGTCTGTGGGTT
HycEup_BamHI	GCGGGATCCACTCTCTTTAATCACGCCGC
HycEdown_BamHI	GCGGGATCCATGGATTATCGTCAGCGTCC
HycEdown_EcoRI	GCGGAATTCTTATTTCAGCGGCGAGTTTTTAC
nuoC_HA_FW_BamHI	GCGGGATCCATGTATCCGTATGATGTGCCGGATTATGCGACCGACTTAACCGCGCAAG
nuoC_RW_BamHI	GCGGGATCCATAATCTTTACGCAGCGGGTG
HycEN+C_FW	ATATACCATGGATTATCGTCAGCGTCC
HycEN+C_RW	CTCCTTAGCTGTCTTTACGCAGCGG
HycEN_RW_EcoRI	GCGGAATTCTTAGTAGGTTTCAGCATCGGTGG
DnuoC_5′_FW	CGCCGACAGTCACCACGGACCATTTGCAATGGTGAACAAT ccatggtccatatgaatatc
DnuoC_3′_RW_KpnI	TGGTCAGCTCAAACGGCGAGAATTCGGTAGCGCGCGCCGGCATggtaccctcct gcgattgtgtaggctggagc
DnuoLM_3′_RW	ACATGTCGATGGCAAGGAACACGCCGATAACGCCGCCCAG gcgattgtgtaggctggagc
nuoC_up_control	CGCATTGCCGTAACTAACC
nuoCD_FW_BamHI	ggatccCACGGACCATTTGCAATGGTG
nuoCD_RW_HindIII	aagcttTTAGCGGTCCACATCTGACATAAC
hycB_FW	GCGAAGCTTGATGAATCGTTTTGTAATTGCTG
hycB_RW	GCGGGATCCTCATTTAGCCTCTCCACTTT
hycF_FW	GCGCTGCAGGATGTTTACCTTTATCAAAAAAG
hycF_RW	GCGGGATCCTCAGATGGCCTCTTTCATATG
tatB_FW	GTGTTTGATATCGGTTTTAG
tatB_RW	TTACGGTTTATCACTCGACG

Separation of the *hycE* gene into two distinct genes on plasmid pHycE was done by the NEBaseChanger method (NEB, Ipswich, MA, USA) using the oligonucleotides HycEN+C_FW and HycEN+C_RW and resulted in plasmid pHycEN+C. This construct was subcloned onto pMAK705 and moved to the chromosome as described 30.

*In trans* complementation was done by cloning the DNA fragment encoding the N‐terminal domain including the connecting alpha helix with AA1‐168 in pJET1.2 (Thermo Scientific, Waltham, MA, USA) using the primers HycEup_XbaI and HycEN_RW_EcoRI.

**Figure 3:**
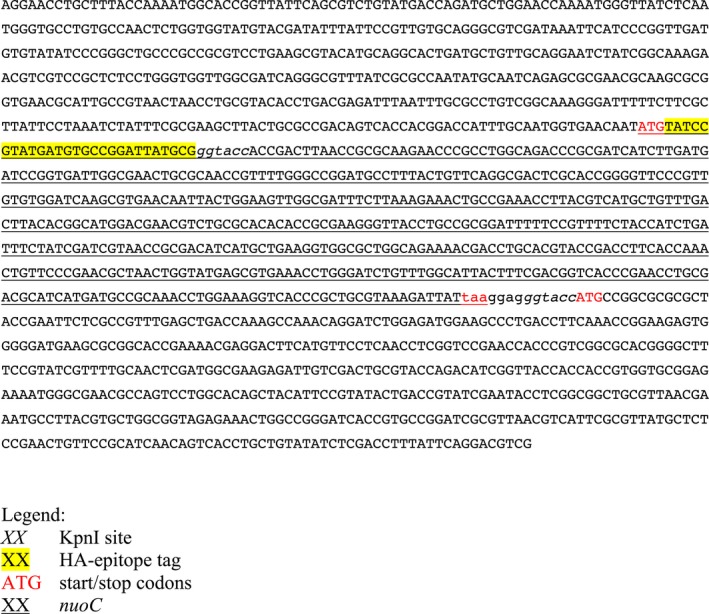
DNA sequence of *nuoC+D* ordered as gBlocks fragment. The 1500‐bp fragment above was obtained from IDT DNA and designed to contain the *nuoC* upstream region, *nuoC* as independent gene (start and stop codons are shown in red; gene is underlined), DNA sequence coding for an included HA‐epitope tag (yellow highlight) and a ribosome binding site for the downstream *nuoD* gene linked to the start codon by a KpnI restriction site (italics).

Strain BW25113 Δ*ndh* has been described before 31. This strain was used for further modification of complex I. Deletion of the DNA sequence encoding the NuoC domain was performed by initially replacing the sequence encoding the domain with the *cat* gene from vector pKD3 (oligonucleotides DnuoC_5′_FW and DnuoC_3′_RW_KpnI) and subsequently removing the resistance cassette using vector pCP20 as described 32, 33. The DNA sequence encoding the downstream NuoD domain received a novel ribosome binding site and start codon. The deletion of the entire region from *nuoC‐L* was performed similarly, but using the oligonucleotides DnuoC_5′_FW and DnuoLM_3′_RW 33. The *nuoC+D* strain was constructed by ordering the corresponding region as gBlocks fragment (DNA sequence in Fig. 3), which contained a stop codon after the codon encoding Y169 in *nuoC*, a ribosome binding site and a start codon for *nuoD* (IDT DNA, Coralville, IA, USA), cloning it as BamHI/BamHI fragment into pMAK705 and introducing it into BW25113 Δ*ndh*.

The pJET‐nuoCD plasmid was constructed by amplification of the chromosomal *nuoCD* gene, including its own ribosome binding site, using the oligonucleotides nuoCD_FW_BamHI and nuoCD_RW_HindIII and Q5 DNA polymerase (NEB) and cloning it blunt‐end into pJET1.2 vector according to the manufacturer's instructions (Thermo Scientific). Expression of the gene is under the control of the *lacUV5* promoter of the vector.

All authenticity of all DNA sequences in the plasmid inserts and strains was verified by sequencing.

### RT‐PCR

The RT‐PCR was performed according to previously published methods 34. RNA was isolated from strains in the exponential growth phase using the SV Total RNA Isolation System according to the manufacturer's instructions (Promega, Fitchburg, WI, USA). An extra DNase I digestion step was performed using RQ1 RNase‐Free DNase (Promega). The RT‐PCR to generate cDNA was performed using M‐MLV Reverse Transcriptase, RNase H Minus and random hexamer primers according to the manufacturer's instructions (Promega). The presence of transcripts was tested with gene‐specific oligonucleotides, which are listed in Table 2, by using 17 PCR cycles to remain within the semi‐quantitative part of the amplification reaction.

### Western blotting and signal quantification

Aliquots of 50 μg total proteins of anaerobically grown cells were separated on a 10% (w/v acrylamide) SDS/PAGE, transferred to a nitrocellulose membrane and challenged with antibodies raised against HycG (1 : 3000, a kind gift from A. Böck/R. G. Sawers) 35, 36. The secondary antibody used was conjugated to HRP enzyme (Bio‐Rad, Hercules, CA, USA), and the light signal generated by the ECL reaction (Thermo Scientific) was recorded with a film. The signal intensities were analysed using imagej software 37.

### Enzymatic assays

Hydrogenase activity was determined as H_2_‐dependent benzyl viologen (BV) reduction using 50 mm MOPS buffer, pH 7.0, with 4 mm BV in a stoppered 1.6‐mL cuvette with 0.8 mL H_2_ gas headspace as described 38. For kinetic determination of FHL activity, cell suspensions were applied to a modified Clark‐type electrode (Oxytherm, Hansatech Instruments, Norfolk, UK), which was equipped with an OXY/ECU module to reverse the polarizing voltage to −0.7 V, where the electrode disc is only sensitive to H_2_ (Hansatech Instruments). A volume of 2 mL degassed MOPS buffer, pH 7.0, was used, and the reaction was started with 15 mm formate. The H_2_ content of the gas headspace of a 15‐mL Hungate tube filled with 7 mL culture was determined by gas chromatography using a GC2010 Plus Gas Chromatograph (Shimadzu, Kyōto, Japan) as described 39.

To measure complex I activity, the NADH‐dependent oxidase activity was determined on a Clark‐type electrode sensitive to O_2_ (Hansatech Instruments) and was performed essentially identical to the H_2_ measurements, with the exception that 1.25 mm NADH was used as substrate 31. Similarly, the succinate oxidase activity was monitored in the presence of 10 mm succinate. Protein concentrations were determined by the method of Ref. 40.

### Sucrose gradient centrifugation

The sucrose gradients were prepared and NADH/ferricyanide oxidoreductase activity was determined basically as described 31. Briefly, membrane extracts from cells grown under the appropriate growth conditions were prepared after cell rupture with an Emulsiflex (EF‐C5; Avestin Europe, Mannheim, Germany) in the presence of PMSF (0.1 mm) and DNase at 160 000 ***g***. The sediment was resuspended in buffer A (50 mm MES/NaOH, 50 mm NaCl) with 1% (w/v) DDM and 5 mm MgCl_2_. After solubilization, the membrane fraction was briefly centrifuged and subsequently applied to a freshly poured 24 mL linear sucrose gradient ranging from 5% to 30% (w/v) sucrose in buffer A supplemented with 5 mm MgCl_2_. After centrifugation for 16 h at 140 000 ***g***, the gradient was fractionated into 24 × 1 mL fractions. The NADH/ferricyanide oxidoreductase activity of 50 μL from each fraction was assayed in 1 mL buffer A containing 1 mm ferricyanide (AppliChem, Darmstadt, Germany). The reaction was started by an addition of 0.2 mm NADH, and the reaction was followed by monitoring the slope at 410 nm in an Ultrospec photometer (Amersham Pharmacia Biotech, Amersham, UK). The rate was calculated using an ε_410_ of 1 mm ^−1^·cm^−1^. Protein concentration was determined by biuret assay 41.

## Results and Discussion

### Formate hydrogenlyase complex retains activity after separation of the N‐ and C‐terminal domains of HycE

In order to investigate the effect of dividing the HycE protein into two separate domains on the activity and stability of the FHL complex, the efficiency of conversion of formate to H_2_ and CO_2_ was quantified by measuring the headspace H_2_ content of the culture after fermentative growth. This activity represents the total yield of H_2_ after growth on glucose medium, but also provides an accurate indication of the activity of the FHL complex, and is particularly useful for assessing low FHL activities. The parental strain produced 7.03 μmol H_2_·mL culture^−1^·OD_600 nm_^−1^, while H_2_ production was basically absent in the negative control strain JW2691, which lacks the *hycE* gene encoding the catalytic subunit HycE. This strain can be partially (71%) complemented for H_2_ production by introducing plasmid pHycE carrying the *hycE* gene. We have previously observed that *hycE* cannot fully complement a Δ*hycE* strain when provided *in trans*, and we assume this to be due to the altered chromosomal context 42. Surprisingly, the Δ*hycE* strain can also be complemented by the plasmid pHycEN+C, which consists of the same insert sequence as on pHycE with the exception that it includes a stop codon within *hycE* (Table 3). This ‘split’, or separated, *hycE* gene in the chromosomal context in strain *hycE*N+C also resulted in H_2_ accumulation to a level similar to that of the parental strain. Strain Δ*hycE*N, which carried a truncation of the N‐terminal domain of HycE, but retained a protein fragment of the C‐terminal domain, was unable to evolve H_2_, a phenotype like that of the *hycE* deletion strain. The Δ*hycE*N strain could be complemented *in trans*, however, by introducing a plasmid encoding the missing N‐terminal portion of HycE, which restored almost 50% of the H_2_ production (Table 3). Complementation could not be achieved by using a fusion of the related NuoC to the C‐terminal domain of HycE. Hence, despite sharing structural similarity, they fulfil distinct roles in both complexes. These assays also showed that the HycE protein, when physically separated into N‐ and C*‐*terminal domains, was as efficient as the native HycE protein in converting formate to H_2_ and CO_2_ during mixed‐acid fermentation.

**Table 3:** Hydrogenase activities and their percentage of the parental activities. The activities were determined from three biological replicates.

Strain (+Plasmid)	H_2_‐headspace quantification (μmol·OD_600 nm_^−1^·mL^−1^)		Formate‐dependent H_2_ evolution (nmol H_2_·mg^−1^·min^−1^)		Total hydrogenase activity H_2_:BV (U·mg^−1^)	
MG1655 (parental)	7.03 ± 0.28	100%	636 ± 28	100%	1.12 ± 0.37	100%
JW2691 (Δ*hycE*)	0.04 ± 0.07	< 1%	1 ± 2	< 1%	0.32 ± 0.10	29%
JW2691 + pHycE	4.99 ± 0.71	71%	190 ± 62	30%	0.56 ± 0.32	50%
JW2691 + pHycEN+C	7.46 ± 1.94	106%	337 ± 173	53%	0.64 ± 0.53	57%
*hycE*N+C	7.23 ± 0.39	103%	350 ± 19	55%	0.67 ± 0.34	60%
Δ*hycE*N	< 0.01	< 1%	1 ± 2	< 1%	0.19 ± 0.05	17%
Δ*hycE*N + pHycEN	3.23 ± 0.19	46%	73 ± 15	11%	0.39 ± 0.18	35%
*nuoC‐hycE*	< 0.01	< 1%	1 ± 2	< 1%	0.19 ± 0.01	17%

In addition to the headspace analysis, which measures accumulation of H_2_ over 16 h, cell suspensions were assayed on a modified Clark‐type electrode with formate as substrate allowing quantitative measurement of the specific activity of the FHL complex. These measurements verified the absence of activity in strains JW2691 (Δ*hycE*), Δ*hycE*N and *nuoC‐hycE*. As could be shown for the H_2_‐headspace measurement, the pHycE and pHycEN plasmids were functional, but were only able to complement the corresponding deletion strains JW2691 (Δ*hycE*) and Δ*hycE*N, to 30% and 11% of the level of the wild‐type strain, respectively (Table 3). While determination of the accumulated H_2_ revealed 100% complementation of H_2_ production, the direct FHL assay led to approximately 50% of the FHL activity rate of the parental strain when the domain‐split HycE protein was introduced either *in trans* on plasmid pHycEN+C in strain JW2691 (Δ*hycE*) or after chromosomal integration in strain *hycE*N+C (Table 2). This result revealed that the two physically separated domains of HycE retain half of the activity of the fused, native protein, despite being fully functional in conversion of glucose during growth. Moreover, the findings demonstrate that providing each component in multiple copies did not result in higher activity, indicating that the amount of the two domains in the complex did not limit the reaction rate.

The samples used in the electrode experiments were subsequently used to generate crude extracts, allowing spectrophotometric quantification of total hydrogenase activity, which is a composite of Hyd‐1, Hyd‐2 and the Hyd‐3 component of the FHL complex. Hyd‐1 and Hyd‐2 are both H_2_‐oxidizing hydrogenases, which allow the cell to scavenge some of the FHL‐produced H_2_. Under these conditions, Hyd‐3 is generally the major contributor to the total hydrogenase activity 42. A fourth hydrogenase, predicted to form a second FHL complex, is usually not expressed under the conditions used in this study and does not contribute to the activity 43, 44. Here, the residual total hydrogenase activity in the Δ*hycE* strain JW2691, representing the combined activities of Hyd‐1 and Hyd‐2, was approximately 30% of that of the wild‐type strain MG1655 (Table 3). Strains Δ*hycE*N and *nuoC‐hycE* showed levels of total hydrogenase activity of 17% of the parental, which, surprisingly, is lower than when only Hyd‐1 and Hyd‐2 are present. Using this assay, the HycE protein separated into two domains complemented to a level of 60% of the wild‐type activity, which was slightly higher than the activity recovered by complementation with a plasmid, pHycE13, encoding the native HycE polypeptide. The formate dehydrogenase H activity, which is the other catalytic component of the FHL complex, followed these trends, but with slightly greater variation (Table 4). This indicates that the formate dehydrogenase polypeptide was able to stably interact with the complex carrying the separated domains of HycE.

**Table 4:** Formate dehydrogenase H activity measured in crude extracts and the percentage of the parental activity. The activities were determined from three biological replicates.

Strain/plasmid	FDH‐H formate:BV (U mg^−1^)	% of parental activity
MG1655	2.61 ± 2.71	100
JW2691 (Δ*hycE*)	0.15 ± 0.24	6
JW2691 + pHycE13	0.03 ± 0.03	1
JW2691 + pHycEN+C	0.43 ± 0.73	16
*hycE*N+C	0.46 ± 0.51	18
Δ*hycE*N	0.14 ± 0.72	5
Δ*hycE*N + pHycEN	0.34 ± 0.54	13
*nuoC‐hycE*	0.14 ± 0.24	5

### Modifications of the HycE N‐terminal domain do not influence transcription of the *hyc *operon

**Figure 4:**
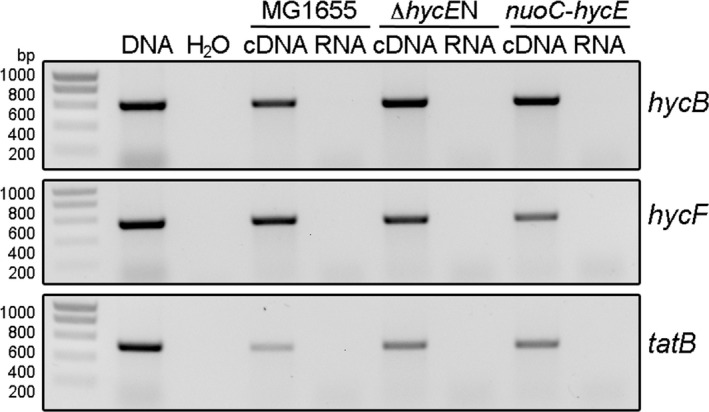
Transcriptional analysis of the *hyc* operon after genomic modifications. RNA was isolated from strains MG1655, Δ*hyc* *E*N and *nuoC‐hycE*, and reverse transcription was performed and subsequently PCR on the cDNA and, for control of DNA contamination, also from the RNA and water. The transcripts that were analysed are *hycB*,* hycF* and *tatB* as labelled on the right side. The first lane shows the size of the correct PCR fragment when obtained from genomic DNA, and the size of the ladder (SmartLadder; Eurogentec, Seraing, Belgium) is shown on the very left.

Bacterial operons often contain internal promoters and regulatory elements 45. In the case of the *hyc* operon, computational evidence predicts a further sigma 70‐dependent promoter in front of *hycI*, the last gene of the operon 46. Therefore, it was investigated whether the genomic modifications in strains Δ*hycE*N and *nuoC‐hycE*, which completely lacked FHL activity, influenced the transcription of the downstream genes. Reverse transcriptase PCR on RNA isolated from those strains in comparison with cDNA obtained from the parental strain MG1655 showed similarly intensive bands for the upstream *hycB* gene, as well as for the downstream *hycF* gene (Fig. 4). Despite this analysis not being quantitative, the overall levels of transcripts were similar, as shown for the transcripts of the constitutive mRNA of the *tatB* gene (Fig. 4). It is concluded, therefore, that the introduced genetic modifications do not alter the transcription of up‐ and downstream genes within the operon.

### Presence of the small subunit protein HycG correlates with the FHL activities

**Figure 5:**
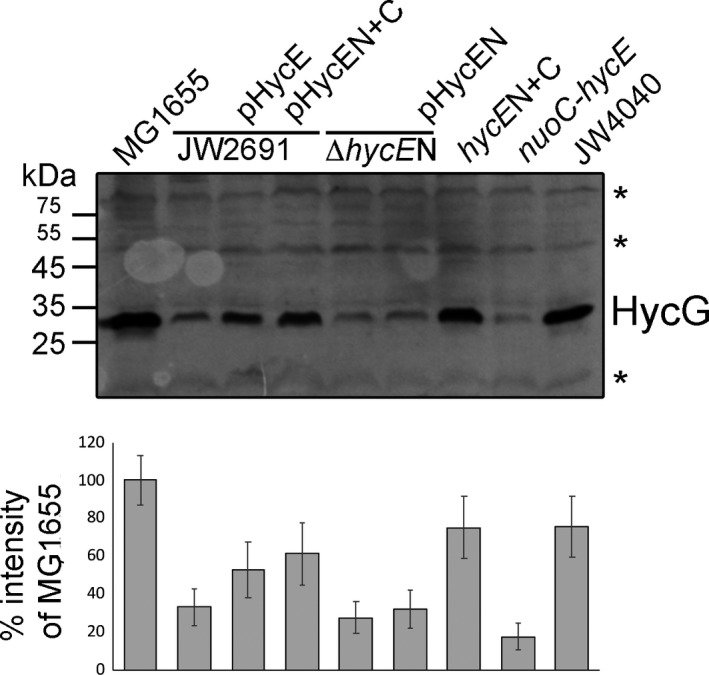
Analysis of HycG levels in HycE variant strains. The amount of HycG protein from anaerobically grown samples was analysed by western blotting using antibodies raised against HycG. A protein amount of 50 μg of strains MG1655, JW2691 (Δ*hycE*), Δ*hyc* *E*N,* hyc* *E*N+C, *nuoC‐hycE* and JW4040 (Δ*fdhF*) was separated by SDS/PAGE either directly or after complementation with the plasmids as indicated. Unspecific and nonhydrogenase related cross‐reactions of the antibody served as an internal loading control and are indicated by an asterisk on the right side of the panel. The histograms in the lower panel show the quantification of the HycG band intensity (±SD) in comparison with the HycG protein amount in MG1655 using imagej.

Analysis of the hydrogenase small subunit HycG of the FHL complex by quantitative western blotting showed that the protein amount is subject to change 8. The absence of HycE in the Δ*hycE* strain JW2691 caused an approximate 70% reduction in HycG levels compared to the wild‐type (Fig. 5). A similar observation for a Δ*hycE* strains was made previously 8. Generally, for hydrogenase assembly to occur, the large subunit must receive its [NiFe]‐cofactor and therefore interact with various delivery proteins and undergo endoproteolytic processing 19, 47. Only then is the protein primed for interaction with its small subunit, and this allows the subsequent initiation of the assembly of the entire FHL complex. However, if processing of the large subunit is delayed or prevented, the small subunit is rapidly degraded 47. This reduced amount of HycG was also observed in the Δ*hycE*N strain, both with and without *in trans* complementation, and the *nuoC‐hycE* fusion strain (Fig. 5). The strains *hycE*N+C and, to a lesser extent, the complementation of Δ*hycE* with pHycE and pHycEN+C show high amounts of HycG, which correlates with their FHL activities. This indicates that the absence of hydrogenase activity, which was observed for some of the HycE variants, correlates with loss of complex integrity and suggests that the N‐terminal domain could be the driving force for assembly of the core FeS‐carrying proteins.

It is notable in this context that the Δ*fdhF* strain JW4040, despite lacking FHL activity due to the absence of the electron‐input subunit FdhH, showed high amounts of HycG (Fig. 5). As FdhH is only loosely associated with the FHL complex, this result indicates that this subunit is not required for the stable assembly of the core FHL complex 27 and that the core FHL complex remains stable even without electron flow through the complex.

### Removal or separation of NuoC causes complete loss of complex I activity

Based on the findings made with HycE, it was decided to test whether the homologous protein of complex I, NuoCD, also revealed similar properties when the two‐domain fusion protein was separated into two distinct proteins. Strain *nuoC+D,* in which the gene encoding NuoCD was physically separated into two genes, was generated. Additionally, the DNA encoding the NuoC domain was deleted in strain Δ*nuoC* to examine the consequences on complex I assembly and activity. All changes were introduced into a strain lacking the *ndh* gene, which codes for the alternative NADH dehydrogenase, and whose presence would interfere with the NADH oxidase activity assay. As a control, a double‐deletion strain Δ*nuoC‐L* was constructed, which, in addition to the Δ*ndh* deletion, was also devoid of most of the genes of the *nuo* operon, including *nuoEFG* encoding the diaphorase domain and part of the membrane domain (*nuoHIJKL*).

The functionality of complex I was initially tested by monitoring the NADH oxidase activity, which is catalysed by complex I and the cytochrome *bo*_*3*_ and cytochrome *bd* oxidases. The strain Δ*ndh,* lacking the alternative NADH dehydrogenase, showed an activity of 0.2 U·mg^−1^ (Table 5), which also has been observed in a previous study 31. The negative control strain lacking complex I (strain Δ*nuoC‐L*) had only a residual oxidase activity of 4%. The two mutant strains Δ*nuoC* and *nuoC+D* had almost no NADH oxidase activity, which indicated a loss of complex I activity (Table 5). A previous study established that deletion of any of the *nuo*‐genes encoding complex I abolished its ability to transfer electrons into the respiratory chain 48. In order to rule out a general effect of the mutations on the respiratory chain, succinate oxidase activity was assessed, which uses succinate dehydrogenase, complex II, instead of complex I to channel electrons into the quinone pool. Succinate oxidase activity was very similar in these strains, ranging from 40 to around 60 mU per mg protein (Table 5), demonstrating that the lack of NADH oxidase activity is specifically due to the absence of complex I.

**Table 5:** NADH and succinate oxidase activity of various *Escherichia coli* complex I deletion strains. The activities were determined from at least two biological replicates.

Strain	NADH oxidase activity (mU·mg protein^−1^)	% Activity of parental	Succinate oxidase activity (mU·mg protein^−1^)
Δ*ndh*	213 ± 12	100	63 ± 14
Δ*ndh,* Δ*nuoC*	3 ± 1	1	50 ± 7
Δ*ndh, nuoC+D*	8 ± 2	4	40 ± 15
Δ*ndh,* Δ*nuoC‐L*	9 ± 8	4	48 ± 7

**Figure 6:**
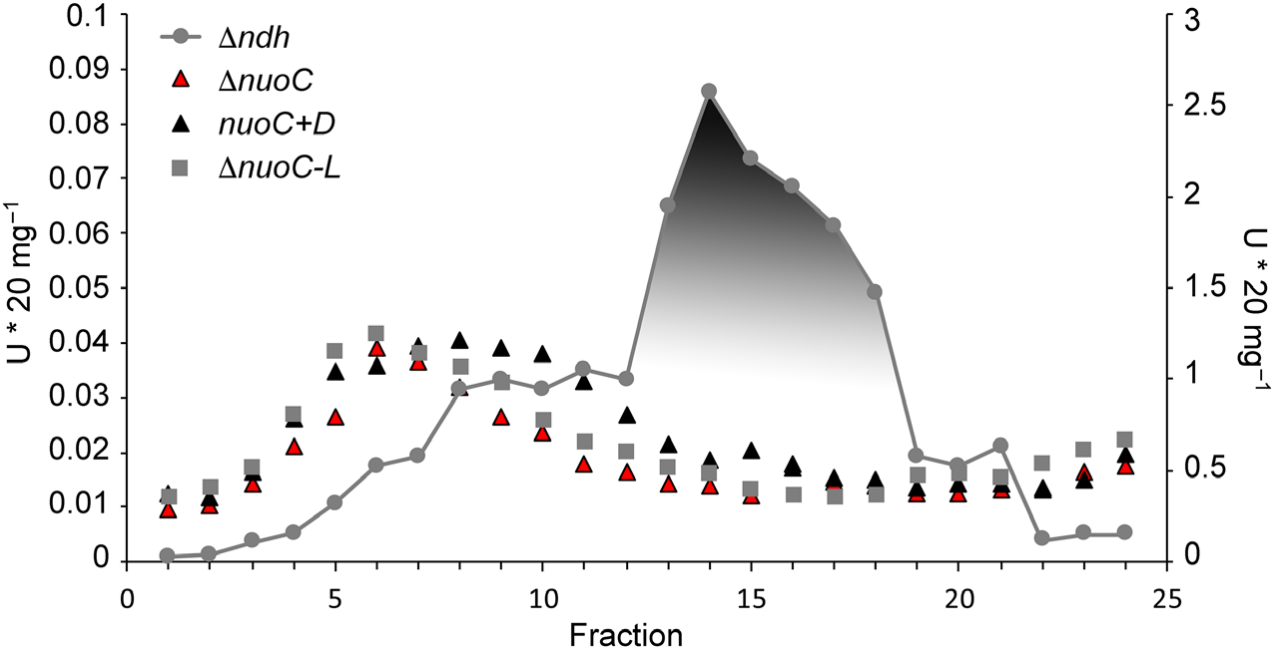
Stability of complex I in various *Escherichia coli* deletion mutants. NADH/ferricyanide oxidoreductase activity of sucrose gradient fractions loaded with detergent extracts. The gradients were normalized to a total load of 20 mg protein. The shaded area represents the distribution of holo‐complex I (scale right). Strains are Δ*ndh* – grey circles with grey connecting line; Δ*nuoC* – red triangles with black border; *nuoC+D* – black triangles; and Δ*nuoC‐L* – grey squares.

The detergent‐solubilized membrane fractions were subjected to a sucrose gradient centrifugation in order to determine the stability and the assembly of the complex in the mutant strains. The fractions were separately assayed for NADH/ferricyanide oxidoreductase activity, which indicates the presence of the FMN‐containing subunit NuoF, which is part of the diaphorase domain of complex I. The activity peaks in fraction 14, corresponding to intact complex I as observed in the Δ*ndh* strain (shaded area in Fig. 6). Complex I mutant strain Δ*nuoC‐L*, which lacks the diaphorase proteins NuoEFG, did not show activity in these fractions. Fractions 5–9 contained a broader, more disperse activity peak, which was originally thought to include the diaphorase activity that had dissociated from the complex. However, because it is also present in the membrane preparations of the Δ*nuoC‐L* strain, this seems unlikely and rather represents an unspecific reaction of the ferricyanide. Furthermore, the activity profile of the extract from strains Δ*nuoC* and *nuoC+D* resembles the negative control, indicating that NuoF is absent.

**Figure 7:**
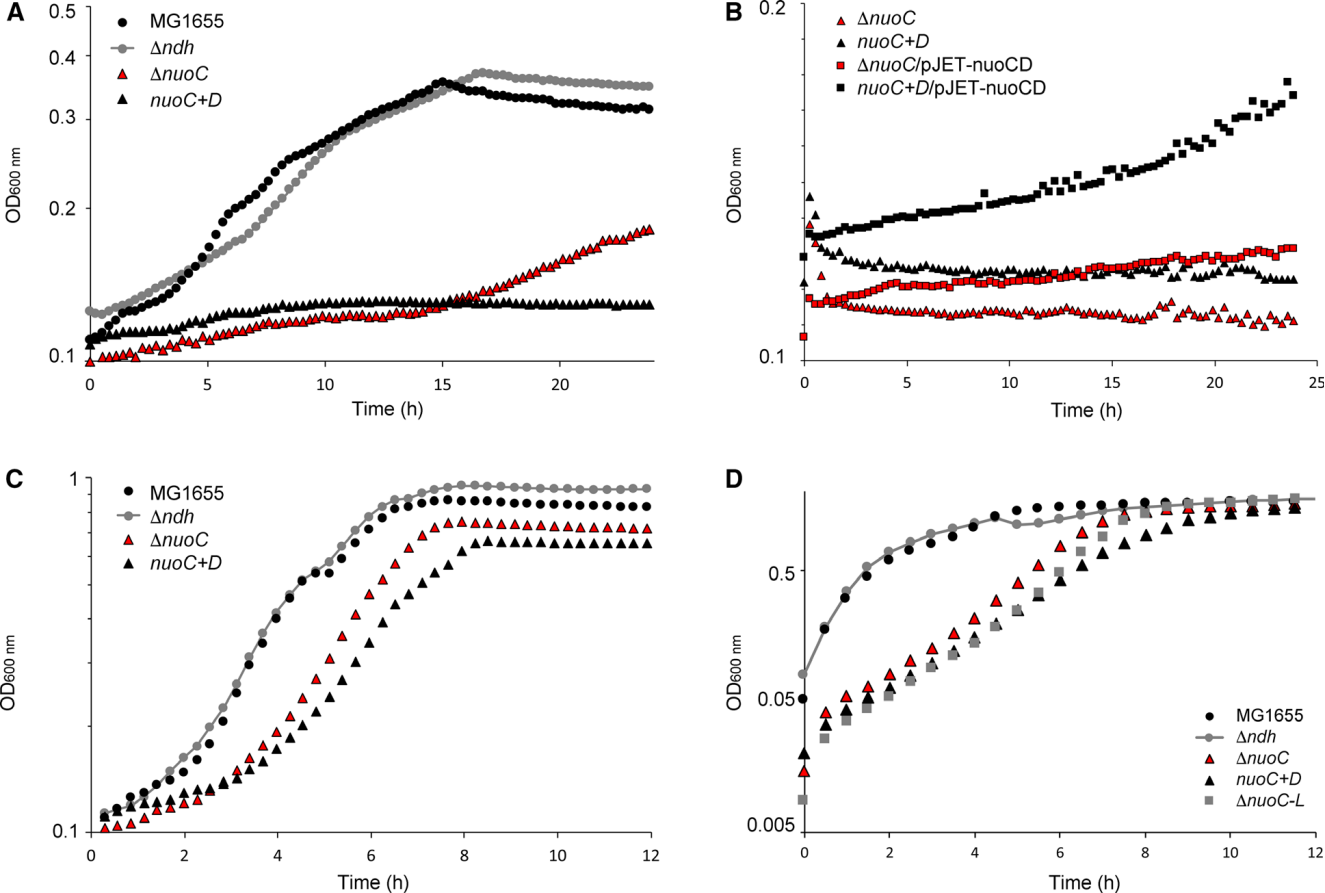
Aerobic growth phenotype of complex I mutants in (A) in acetate minimal medium (B) after complementation (C) glucose minimal medium and (D) glycerol/LB medium. All growth curves were recorded in a plate reader with 150 r.p.m. shaking in 150 μL volume. The graphs represent the average of three biological triplicates. For all graphs: BW25113 – black circles; Δ*ndh* – grey circles with grey connecting line; Δ*nuoC* – red triangles with black border; *nuoC+D* – black triangles; and Δ*nuoC‐L* – grey squares. For panel 7B only: Δ*nuoC*/pJET‐nuoCD – red squares with black border; and *nuoC+D*/pJET‐nuoCD – black squares.

Assuming that the mutations could destabilize complex I after extraction from the membrane, but nevertheless allowed retention of a residual activity in whole cells, the strains were subjected to growth experiments in an *in vivo* assay (Fig. 7A). The cells were grown aerobically with acetate as sole nonfermentable carbon source, which requires activity of complex I in order to maintain the NADH/NAD^+^ ratio within the cells. This in turn is necessary for the function of the TCA cycle and the glyoxylate shunt to produce amino acids 49. The growth monitoring during 24 h showed that the Δ*ndh* strain had no growth defect compared to the parental strain, as observed before 31. In contrast, both the Δ*nuoC* strain and the *nuoC+D* strain did not grow within the first 16 h of the experiment, while eventually the Δ*nuoC* strain showed a slight increase in cell density (Fig. 7A). The absence of growth with acetate, as in the Δ*nuoC* and *nuoC+D* strains, typically indicates lack of complex I activity. A complementation of the strains Δ*nuoC* and *nuoC+D* with a plasmid encoding the *nuoCD* gene, including its ribosome binding site, was attempted (Fig. 7B). However, the growth of the Δ*nuoC* or *nuoC+D* strain on acetate‐containing minimal medium could only be restored to a small degree with the *nuoCD* plasmid, attaining optical densities of 0.18 (*nuoC+D*), while the parental strain grew to an OD of 0.33. This could indicate a dominant phenotype of the chromosomal *nuoCD* modification; however, it also does not entirely exclude effects caused by disruption of the *nuo* operon. All strains were in parallel subjected to growth in glucose medium, where they exhibited no difference in growth behaviour (Fig. 7C). Furthermore, glycerol medium was also tested, where the strains still attained the same final optical density in the stationary phase (Fig. 7D). The Δ*ndh* strain and its BW25113 parental derivative grew very similarly, with a growth rate μ of 1.45 and 1.74 h^−1^, respectively. In contrast, the strains Δ*nuoC*,* nuoC+D* and Δ*nuoC‐L* had significantly, but similarly, diminished growth rates of 0.54, 0.46 and 0.52 h^−1^, respectively.

Therefore, all of the growth experiments indicate that the *nuoC* deletion and separation of *nuoCD* into two genes abolish complex I activity in whole cells in *in vivo* experiments. The absence of activity in the Δ*nuoC* strain was not surprising, because even single amino acid exchanges in NuoC had a similar effect 26. What was surprising, however, was the different effects of separating the paralogous genes into two parts in the case of FHL and complex I. The FHL complex tolerated the introduced changes, while complex I presumably did not assemble anymore. The region within the proteins where separation of the domains occurred was based on protein alignments and the presence of the Met153 residue in HycE, which was directly hijacked as initiation codon (see alignment in Fig. 2). However, the amino acid sequence in this region is not as conserved as other regions of the protein, and without further structural information of HycE and NuoCD, it is very difficult to predict the location of the loop region and its function in connecting the modules of the complexes.

## Conclusions

The research conducted here on the function of the N‐terminal domains of the HycE and NuoCD subunits of the *E. coli* FHL complex and complex I, respectively, reveals that these domains have been evolutionarily conserved and appear to have an important role in complex assembly. It is still not understood what the regulatory steps in the assembly of these large complexes are, but it is reasonable to assume that assembly must involve a highly ordered sequence of events, where accumulated subcomplexes represent intermediate steps 12, 50. The FeS proteins tend to be unstable in the absence of other components, and thus, the nucleation of the core complex (HycB, E, F, G or NuoB, CD, H, I, L) probably starts with the catalytic subunit HycE and its paralogue NuoCD, respectively. These subunits reside next to the membrane interface, while the formate dehydrogenase or NuoEFG proteins represent the last complex‐specific steps in the separate evolution of the two complexes and are located further away from the membrane 12. We identified the N‐terminal domains of the HycE and NuoCD proteins as key determinants in initiating the assembly of this core complex. The different effects of splitting the proteins in two parts show that despite the existing phylogenetic relationship between the FHL and complex I, the latter tolerates splitting of the *nuoCD* gene less well.

## Conflict of interest

The authors declare no conflict of interest.

## Author contributions

PS and SB investigated complex I activity; UL, CB and CP investigated FHL activity; TF and CP supervised the study, provided resources and validated the results; and CP wrote the original draft.
